# Sero-prevalence of HBsAg in naive HIV-infected patients in a rural locality of Cameroon

**DOI:** 10.1186/s13104-018-3159-2

**Published:** 2018-01-16

**Authors:** Jean-Patrick Molu, Marie Chantal Ngonde Essome, Chavely Gwladys Monamele, Richard Njouom

**Affiliations:** 1 Laboratoire de Biologie Humaine, Centre de Recherches Médicales, Institut de Recherches Médicales et d’Etudes de Plantes Médicinales, PO Box 13033, Yaounde, Cameroon; 2 Yaounde University Teaching Hospital, PO Box 1634, Yaounde, Cameroon; 3 Department of Microbiology and Parasitology, University of Buea, PO Box 63, Buea, Cameroon; 4 Centre Pasteur of Cameroun, PO Box 1274, Yaoundé, Cameroon

**Keywords:** Co-infection, HIV, HBV, Prevalence, Rural locality, Mfou, Cameroon

## Abstract

**Objective:**

This study was performed in order to fill the gap of knowledge regarding sero-epidemiology of hepatitis B virus (HBV) amongst Human Immunodeficiency virus (HIV)-infected patients and to assess the risk factors associated with HBV co-infection in a rural locality of Cameroon. A retrospective and cross-sectional study was carried out from January 2008 to April 2014 within the Mfou District Hospital. Naive HIV-infected patients were enrolled in the study and tested for hepatitis B surface antigen (HBsAg). Preliminary pre-therapeutic data essential for follow-up was collected from the participants.

**Results:**

Overall, the sample size was constituted of 712 HIV-infected patients. The prevalence of HBsAg was 8.99%. A significant difference was observed in the proportion of HBsAg positive subjects with respect to the year of inclusion; higher proportions were observed between 2011 and 2014 (*P*-value = 0.007). Majority of HBV co-infected participants had severe immuno-suppression with CD4 counts lower than 100 cells/µL as compared to HIV mono-infected population but the difference was not statistically significant. Our results confirm the high prevalence for HBV infection among HIV-infected patients in the Mfou District Hospital. These findings will enable stake holders to be better armed in the elimination of viral hepatitis as a public health problem.

## Introduction

In 2016, the World Health Assembly adopted the first “Global Health Sector Strategy on Viral Hepatitis 2016–2021” [1]. The strategy has a vision of reducing new viral hepatitis infections by 90% and reducing deaths due to viral hepatitis by 65% by 2030. This target can only be possible if there is in-depth knowledge of the sero-epidemiology of HBV particularly in high-risk groups as well as in rural regions where there is limited data on HIV-HBV co-infection. A systematic review of the epidemiology of HIV co-infection with HBV in sub-Saharan Africa reported hepatitis B surface antigen (HBsAg) prevalence, of up to 20% in HIV infected patients in Cameroon [2]. A few other studies have reported sero-prevalences ranging from 8 to 25.5% [3–7]. This study was performed in order to fill the gap of knowledge regarding sero-epidemiology of HBV amongst HIV-infected patients in Mfou, a rural locality of Cameroon.

## Main text

### Methods

#### Study population

We carried out a retrospective and cross-sectional study from January 2008 to April 2014. It took place within the Mfou District Hospital (MDH), a hospital setting for monitoring HIV-infected subjects found in a rural zone of Cameroon. Preliminary information regarding basic biological parameters was collected from the patient records prior to antiretroviral treatment initiation.

#### Sample collection and laboratory analysis

Blood samples were collected from all participants into a 5 mL dry tube and a 5 mL EDTA tube. Following collection, the dry tube was centrifuged at 2500 rpm for 15 min in order to collect serum required for subsequent analyses. Since all enrolment was made in MDH upon initiation of anti-retroviral therapy, all participants had prior results for HIV testing. However, a novel detection of HIV in the serum of the subjects was carried out by the HIV-½ rapid diagnostic tests (Determine™ HIV-½, Alere Medical Co., Japan), an immunochromatographic assay and subsequently on ImmunoComb^®^ II Anti-HIV 1&2 EIA kit (Orgenics Ltd, Yavne, Israel) for qualitative and differential detection of antibodies to HIV types 1 and 2. The CD4 lymphocyte counts were detected on whole blood collected in EDTA tube using a Partec CyFlow^®^ automation system. Liver transaminases on the other hand were measured on the participant’s serum with a mono-reagent kit (Biolabo SAS, France) and run in a spectrophotometer. According to the kit, normal levels of alamine amino transferase (ALAT) and aspartate amino transferase (ASAT) were between 8 and 35 IU/L. Detection of HBsAg was carried out by immuno-chromatographic method with HBV One Step Rapid Assay (DiaSpot^®^, Jawa Barat, Indonesia). HIV and HBsAg serologic assay were validated with known in-house positive and negative controls. All laboratory analyses were performed at the MDH following the manufacturer’s instructions.

#### Statistical analysis

Data obtained were processed in Excel and statistical analysis performed with SPSS version 22.0. Results were interpreted by the Chi square test with a degree of significance (P) at the 5% threshold.

### Results

A total of 712 HIV-infected patients were enrolled in MDH from 2008 to 2014. Table 1 summarizes the characteristics of the study population. The mean age of the study population was 38.90 years with an age range of 4–93 years. Similarly the mean values for CD4 counts, ASAT and ALAT were respectively, 187.17 (range 6–960), 31.99 (range 0.65–287), and 22.16 (range 0.51–202).

**Table 1:** Characteristics of study population

	N	Minimum	Maximum	Mean	Standard deviation
Age (years)	712	4	93	38.90	10.56
CD4 counts (cells/µL)	712	6	960	187.17	127.85
ASAT (IU/L)	708	0.65	287	31.99	24.37
ALAT (IU/L)	712	0.51	202	22.16	20.69

**Fig. 1:**
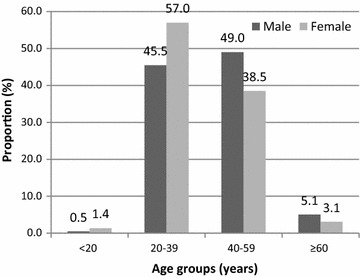
Age and sex distribution of the study population

The study population was predominantly female, with a sex ratio of 2.59. The 20-40 years age group was the most affected population among women whereas the 40–60 years age group was the most represented in males (Fig. 1). The least represented age group was the < 20 years.

Overall, there were 64 participants positive for HBsAg (8.99%; CI 7–11%). Table 2 summarizes the association between HIV-mono infected subjects and HIV-HBV co-infected subjects with respect to socio-demographics and biological parameters. The only significant association observed between the two groups was with respect to the year of inclusion; higher proportions were observed between 2011 and 2014 (*P* = 0.007). The 20–39 years age group was the most predominant in both population followed by the 40–59 years age group. Majority of HBV co-infected participants had severe immuno-suppression with CD4 counts lower than 100 cells/µL as compared to HIV mono-infected population where CD4 counts between 200 and 499 cells/µL predominated. A noticeable high proportion of participants had liver transaminase levels ≥ 2N (26.2% for ALAT and 39% for ASAT) among the HIV mono-infected population as compared to 14.1 and 28.1%, respectively in the HBV co-infected population though the relationship was not significant.

**Table 2:** Characteristics of study population with respect to HBV status

	HIV mono-infected N (%)	HBV co-infected N (%)	P-value
Sex			0.953
Male	180 (27.8)	18 (28.1)	
Female	468 (72.2)	46 (71.9)	
Age group (years)			0.610
< 20	7 (1.1)	1 (1.6)	
20–39	345 (53.2)	38 (59.3)	
40–59	271 (41.8)	24 (37.5)	
≥ 60	25 (3.9)	1 (1.6)	
Year of inclusion			0.007
2008	75 (11.6)	4 (6.3)	
2009	67 (10.3)	3 (4.7)	
2010	108 (16.7)	3 (4.7)	
2011	129 (19.9)	15 (23.4)	
2012	109 (16.8)	12 (18.8)	
2013	124 (19.1)	20 (31.3)	
2014	36 (5.6)	7 (10.9)	
CD4 counts (cells/µL)			0.365
< 100	188 (29.0)	24 (37.5)	
100–199	176 (27.2)	17 (26.6)	
200–499	273 (42.1)	21 (32.8)	
≥ 500	11 (1.7)	2 (3.1)	
ALAT (IU/L)			0.056
N	478 (73.8)	55 (85.9)	
2N	134 (20.7)	6 (9.4)	
> 2N	35 (5.5)	3 (4.7)	
ASAT (IU/L)			0.221
N	393 (61.0)	46 (71.9)	
2N	196 (30.5)	14 (21.9)	
> 2N	55 (8.5)	4 (6.2)	
Total	648	64	

### Discussion

This study laid more emphasis on the high endemicity of HBV in Cameroon as previously reported [8]. The HBsAg prevalence in HIV infected individuals was 8.99%, which is comparable to the 12.6% obtained by Zoufaly et al. among patients initiating ART in rural Cameroon [5]. Similarly high proportions were obtained by several authors in Cameroon; 11.8% in an investigation of overt and occult hepatitis-B infection among HIV-infected individuals [9], 9.8% among ART-treated patients [4], the 7.85% among HIV-infected pregnant women [6], and 25.5% in the South West and Littoral regions [7]. One of the most probable causes of the high incidence of HBV/HIV co-infection is the fact that both viruses have similar routes of transmission [10].

The significant association observed between the presence of HBsAg and the enrolment periods shows the increasing trend of HBV infection among HIV-positive patients. This is a call for concern since there is a recommended vaccine that protects against HBV infection and induces protective antibodies in 95% of individuals that complete their vaccination series [8]. Cameroon included the hepatitis B vaccine in its expanded program of immunization (EPI) in 2005 and it is the probable reason for the low circulation of HBsAg among the young population. Targeted education and vaccination campaigns should however be performed to provide immunization among the young adults which was the most represented age group in this study and the most affected by HBV co-infection.

On average, there were at least twice as more women than men in our study population. This can be explained by the fact that most women get screened for HIV during pregnancy as part of their pre-natal examination in order to curb mother to child transmission. As reported by previous studies, gender was not a risk factor for HBV infection (*P* = 0.953) [3, 11–13]. Conversely, Diwe et al. in Nigeria reported that men were at higher risk of HBV infection than women due to sharing of injection equipments amongst drug addicts [14].

Overall, more than 50% of the study population had CD4 counts below 200 cells/µL and thus indicates late diagnosis of HIV in these patients. These late diagnoses represents missed opportunities for treatment and prevention, and consequently results in poor prognosis of disease and increased likeliness for co-morbidities to develop. This study however noted no statistical significance between the degree of immune-suppression as reflected by CD4 counts and HBV co-infection. Previous findings from Cameroon relate lower CD4 counts to increased risk of HBV co-infection [15].

Liver transaminases were more raised in HIV mono-infected population as compared to co-infected population. Despite the fact that this association was not significant, these findings contradict several reports where higher hepatic cytolysis was observed in HBV co-infected patients compared to those with HIV mono-infection [16]. This is probably due to the fact that most co-infected patients were either recently infected or were still at the stage of inactive hepatitis when no liver cytolysis had yet occurred [17].

### Conclusion

Our results confirm the high prevalence for HBV infection among HIV-infected patients in the MDH though no risk factors were associated to HBV co-infection. These findings will enable stake holders to be better armed in targeting the “Global Health Sector Strategy on Viral Hepatitis 2016–2021” vision of eliminating viral hepatitis as a public health problem [1].

## Limitations

- This study did not enable description of socio-demographic risk factors associated with both viral infections for a better understanding of results obtained.
- Information regarding the stage of HIV would have been essential to study as risk factor for HBV infection.
- Information on HIV viral load, an essential parameter for HIV monitoring was lacking.
- HBsAg was detected at one point of the study and did not enable to distinguish between acute HBV infection which might eventually resolve from chronic viral hepatitis [8].

## Abbreviations

HBV: hepatitis B virus
HIV: human immunodeficiency virus
HBsAg: hepatitis B surface antigen
MDH: Mfou District Hospital
ALAT: alamine amino transferase
ASAT: aspartate amino transferase
